# The Danish 22q11 research initiative

**DOI:** 10.1186/s12888-015-0594-7

**Published:** 2015-09-17

**Authors:** Henriette Schmock, Anders Vangkilde, Kit Melissa Larsen, Elvira Fischer, Michelle Rosgaard Birknow, Jens Richardt Møllegaard Jepsen, Charlotte Olesen, Flemming Skovby, Kerstin Jessica Plessen, Morten Mørup, Ollie Hulme, William Frans Christiaan Baaré, Michael Didriksen, Hartwig Roman Siebner, Thomas Werge, Line Olsen

**Affiliations:** 1 Institute of Biological Psychiatry, Mental Health Centre Sct. Hans, Copenhagen University Hospital, Boserupvej 2, DK-4000 Roskilde, Denmark; 2 The Lundbeck Foundation Initiative for Integrative Psychiatric Research, iPSYCH, Aarhus and Copenhagen, Denmark; 3 Danish Research Centre for Magnetic Resonance, Centre for Functional and Diagnostic Imaging and Research, Copenhagen University Hospital Hvidovre, Kettegaard Allé 30, DK-2650 Hvidovre, Denmark; 4 DTU Compute, Department of Applied Mathematics and Computer Science, Technical University of Denmark, Richard Petersens Plads Building 324, DK-2800 Kgs Lyngby, Denmark; 5 H. Lundbeck A/S, Ottiliavej 9, DK-2500 Valby, Denmark; 6 Child and Adolescent Mental Health Center, Copenhagen University Hospital, Mental Health Services, Capital Region of Denmark, Bispebjerg Bakke 30, 2400 København NV, Denmark; 7 Lundbeck Foundation Center for Clinical Intervention and Neuropsychiatric Schizophrenia Research (CINS) and Center for Neuropsychiatric Schizophrenia Research (CNSR), Copenhagen University Hospital, Mental Health Services, Capital Region of Denmark, Ndr. Ringvej 29-67, DK- 2600 Glostrup, Denmark; 8 Department of Pediatrics, Aarhus University Hospital, Norrebrogade 44, DK-8000 Aarhus C, Denmark; 9 Department of Clinical Genetics, Copenhagen University Hospital, Blegdamsvej 9, DK-2100 Copenhagen, Denmark; 10 Department of Clinical Medicine, University of Copenhagen, Blegdamsvej 3B, DK-2200 København N, Denmark; 11 Department of Neurology, Copenhagen University Hospital Bispebjerg, Bispebjerg Bakke 23, 2400 København NV, Denmark

## Abstract

**Background:**

Neurodevelopmental brain disorders such as schizophrenia, autism and attention deficit hyperactivity disorder are complex disorders with heterogeneous etiologies. Schizophrenia and autism are difficult to treat and often cause major individual suffering largely owing to our limited understanding of the disease biology. Thus our understanding of the biological pathogenesis needs to be substantiated to enable development of more targeted treatment options with improved efficacy. Insights into the pre-morbid disease dynamics, the morbid condition and the underlying biological disease mechanisms may come from studies of subjects with homogenous etiologies. Breakthroughs in psychiatric genetics have shown that several genetic anomalies predispose for neurodevelopmental brain disorders. We have established a Danish research initiative to study the common microdeletion at chromosome 22q11.2, which is one of the genetic anomalies that confer high risk of schizophrenia, autism and attention deficit hyperactivity disorder.

**Methods/design:**

The study applies a “cause-to-outcome” strategy to identify pre-morbid pathogenesis and underlying biological disease mechanisms of psychosis and secondarily the morbid condition of autism and attention deficit hyperactivity disorder. We use a population based epidemiological design to inform on disease prevalence, environmental risk factors and familial disposition for mental health disorders and a case control study design to map the functional effects across behavioral and neurophysiological traits of the 22q11 deletion in a recruited sample of Danish individuals.

**Discussion:**

Identification of predictive pre-morbid clinical, cognitive, functional and structural brain alterations in 22q11 deletion carriers may alter current clinical practice from symptomatic therapy of manifest mental illness into early intervention strategies, which may also be applicable to at risk subjects without known etiology. Hopefully new insights into the biological disease mechanisms, which are mandatory for novel drug developments, can improve the outcome of the pharmacological interventions in psychiatry.

## Background

Neurodevelopmental brain disorders, such as schizophrenia, autism and attention deficit hyperactivity disorder (ADHD) are major causes of life-long suffering to the individual and impose huge costs to society. Emerging evidence within the psychosis research field suggest that early intervention may improve treatment outcome and even reduce the disease transition rate [1], yet treatment options are poor at best. This is largely due to the insufficient understanding of the early prodromal traits and the disease etiology and pathology.

Recent human genetic studies have reported that deletions or duplications of chromosomal regions termed copy number variants can cause a wide range of neurodevelopmental brain disorders [2–14]. An emerging feature of these findings is that a single disorder can be caused by different copy number variants (i.e. *genetic heterogeneity*) and that a single copy number variant can confer risk of different disorders (i.e. *pleiotropy*). The discoveries have increased the understanding of developmental mental disorders as arising from complex patterns of etiologies and pathologies that are not confined to the boundaries of the purely conventional diagnostic systems of current psychiatry. Thus, diagnostic homogeneity is no guarantee for uniform pathology and even less so, for shared etiology.

We believe that the findings of genetic high risk factors for mental disorders motivate clinical “cause to outcome” studies, focused on subjects who share a common genetic risk factor for whom the observed symptomatology and underlying biological perturbations can be ascribed to that particular risk factor.

The 22q11.2 microdeletion on the long arm of chromosome 22 is the most common copy number variant in humans, with an estimated prevalence of approximately 1:2000 to 1:4000 live births [15–17]. The majority (approximately 90 %) of microdeletions that have been detected at chromosome 22q11.2 are 3 megabases in size. A 1.5 megabases deletion nested within the common 3 megabase region is less frequently observed [18] and rare atypical microdeletions of different sizes have also been described [19–21]. The common deletion was recognized early on as the cause of DiGeorge Syndrome and velo-cardio-facial syndrome (and recently the 22q11 deletion syndrome) encompassing multiple somatic conditions, including congenital heart disease, velopharyngeal insufficiency, immune deficiency, facial dysmorphism and intellectual disability [22–24].

Over the past decades it has become increasingly clear that the common 22q11 deletion also confers very high risk of neurodevelopmental brain disorders, including schizophrenia, autism, and ADHD. Studies of patients with schizophrenia find that carriers of the deletion account for approximately 1–2 % of the sporadic cases [25]. The International Consortium on Brain and Behavior in 22q11.2 has recently provided the best estimates of disease prevalence so far. Their data show that schizophrenia (in broad sense) affects 24 % of the 22q11 deletion carriers during adolescents and 41 % in adulthood. Among the children and adolescents 12 and 27 %, respectively, have been diagnosed within the autism spectrum disorders, while a diagnosis of ADHD has been given to 37 % of the affected children, 24 % of the adolescents and 16 % of the 22q11 deletion carriers who are in their early adulthood [26].

Widespread cognitive deficits and neuroanatomical and neurophysiological alterations are prevalent in 22q11 deletion carriers. The cognitive impairments span across several domains of neurocognitive functions including intelligence, spatial and verbal memory, working memory, problem solving and planning, attention, processing speed, impulsivity, to social cognitive functions (i.e. emotion recognition and Theory of Mind), and social behavior [27–29]. The neuroanatomical phenotype include delayed brain maturation during adolescence [30], and regional and whole brain alterations such as significant reductions in regional brain volume, cortical thickness, gyrification, and diminished grey - and white matter density, as well as alterations in functional connectivity in adulthood [31–33]. Furthermore, 22q11 deletion carriers have shown neurophysiological alterations during resting-state electroencephalography (EEG) in the expression of functional microstates defined by few recurrent and dominant classes of scalp topographies, which suggests that the basic resting state condition in 22q11 deviates from the one reported in control subjects [34]. Also reduced processing abilities in response to auditory mismatch negativity (MMN) stimuli have been reported [35].

Longitudinal studies in 22q11 deletion carriers have primarily focused on psychosis outcome and have outlined several indicators of increased risk of transition to psychosis, including low verbal IQ [36, 37], poor theory of mind abilities and reduced processing speed [27]. Regional reductions in grey matter volume of the temporal cortex [38] and right medial orbitofrontal cortex [39] have been associated with positive symptom severity. Reduced functional activation of intraparietal sulcus during working memory testing have been associated with more severe clinical symptoms of unusual thought content and delusional ideas in 22q11 deletion carriers [40]. Sinderberry and collaborators merged multiple data across cognitive functions and brain anatomy as well as facial morphology and were able to identify two subgroups of 22q11 carriers that were not defined by clinical diagnostic criteria [41]. The diverging phenotype sub-groups may reflect different developmental trajectories but are not sufficiently specific in their ability to identify subjects at risk of mental disorders. Hence, they cannot be implemented in early intervention strategies.

### Rationale

Prompted by these findings, we have established the National Danish 22q11 Research Initiative to extend the existing knowledge of the 22q11 deletion as an at-risk model for neurodevelopmental disorders. The rational for doing focused studies on one genetic risk factor such as the 22q11 deletion stems from the etiological homogeneity i.e. all cases are affected by a the same high impact risk factor. The assumption is that the pathogenesis and pathology caused by one risk factor is less heterogeneous than within diagnostic group of mental disorder in general where multiple etiologies are at play. Therefore, the power to detect significant effects of the 22q11 deletion on symptomatology is likely to be higher compared to traditional high-risk studies focusing on a single diagnosis. Thus, we can track the path from the 22q11 deletion across biological and functional modalities to the pathophysiological outcomes and clinical presentations.

### Aim

The overall objective is the identification of a multi-dimensional model, which spans cognitive, neuroanatomical, and neurophysiological domains as wells as environmental exposures and family disposition and is predictive of the pathology currently defined by the diagnostic criteria for schizophrenia, autism or ADHD.

## Methods

### Overall design

In order to achieve our overall objective, we are applying two overall complementary approaches: a population based registers study and a functional and structural study.

We adopt a population based epidemiological design to *(a)* inform on the incidence rate of developmental mental disorders in a nation-wide population of 22q11 deletion carriers in Denmark, *(b)* map family disposition of mental disorders, environmental factors and prenatal stressors (e.g. infections and fatigue) and address the impact of these factors on disease outcomes, and *(c)* to follow the trajectories in disease propagation over time.

The functional and structural studies are based on a recruited sample of 22q11 deletion carriers. In these studies we use (a) a cross-sectional case–control study design to identify genuine imprints of the 22q11 deletion itself, *(b)* a case-only design nested within the cross-sectional setup to uncover correlations between cognitive and neuro- anatomical and -physiological measurements and levels of psychopathology, *(c)* a family design to investigate the familial resembles of inherited forms of the 22q11 deletion to discover additional risk factors/biomarkers.

### Epidemiological register based sub-studies

Incidence rates of disease and the impact on disease outcomes of early life events and environmental exposures are difficult to estimate from observational studies in humans. We apply epidemiological survival analyses to mine the wealth of health information in the Danish registries to address these issues for a nation-wide sample of 22q11 deletion carriers compared to the Danish population as a whole.

In Denmark all citizens are registered with a unique civil registration number given at birth or upon immigration. The civil registration number is registered in the Danish civil registry, which holds information on all persons who have lived in Denmark since April 2nd 1968. The Danish health registries automatically obtain records on all clinical discharge diagnoses defined according to the International Classification of Diseases (ICD) system and the information is linked to the civil registration number. Thus the national health registries in Denmark hold information on the disease propensity of the Danish population including information on socioeconomic factors, family history, and urbanization etc.

The epidemiological studies utilize information obtained from the National Danish Health Registries including the Danish Civil Registration System, the Danish National Patient Registry, the Danish Psychiatric Central Register and the Danish Cytogenetic Central Register.

The diagnostic information in the Danish health registries refers to the ICD-8 protocol until 1994 whereas ICD-10 criteria have been applied from January 1st 1994 to date. The Psychiatric Central Register includes information on all psychiatric inpatient facilities since April 1, 1969 and in addition for outpatient facilities since 1995. The Danish Cytogenetic Central Register holds information on all Danish subjects who have been referred for clinical genetic testing on clinical indication and have been tested positive for a genetic anomaly [42]. Clinical genetic testing for deletions within the chromosome 22q11.2 region was implemented in Denmark in 1994. The genetic testing is solely performed on clinical indication and is not mandatory. The reporting of information to Danish Cytogenetic Central Registry is self-imposed by all departments performing clinical genetic tests in Denmark, and the registry is administered by representatives from these departments.

### Subjects

**Fig. 1:**
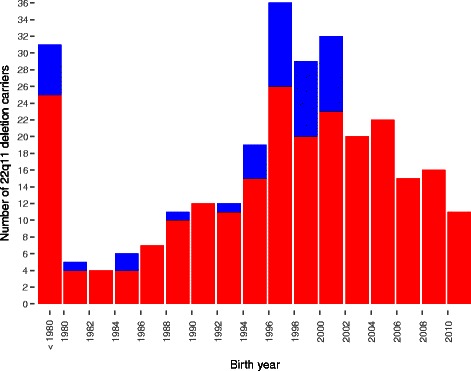
Birth year distribution of all known Danish carriers of the chromosome 22q11.2 deletion and a recruited sub-sample of 44 individuals. Figure subscript. The number of known carriers of the 22q11 deletion born each year is depicted in red while the distribution of the recruited sub-sample is shown in blue

The study population contains all Danish citizens born in 1954 to present. A total number of 244 Danish citizens (125 males and 119 females) are recorded in Danish Cytogenetic Central Register as 22q11 deletion carriers. This group of known Danish 22q11 carriers represents those that have been referred for genetic testing based on some clinical indication and have consent to be tested. The birth year distribution of the known Danish 22q11 carriers is shown in red in Fig. 1. The majority (60 %) of the known 22q11 deletion carries have been tested positive for the 22q11 deletion before their 6th years birthday and the median age of a positive genetic test result is 5 years (age range 0–45 years).

### Inclusion criteria

All Danish citizens born in Denmark after 1954 or later who have references to both parents in the Danish civil register.

### Exclusion criteria

Danish citizens not born in Denmark or who have migrated out of the country or who do not have references to both parents in the Danish civil register.

### Data retrieval

For each person, who fullfills the inclusion criteria, the first psychiatric diagnosis (e.g. schizophrenia) is derived from Danish Psychiatric Central Register and the date for a positive 22q11 deletion test result is obtained from the Cytogenetic register. Information on age, gender, birthplace, familial disposition and somatic diagnoses are retrieved from the Danish National Patient Registry. We will follow the cohort from a given age (where the ‘at risk’ for developing a given mental disorder is present), until the date of their first diagnosis for this given disorder, the date of death, the date of emigration/disappearance or 1 January 2015, whichever comes first. We will use survival analyses (i.e. Poisson regressions), with the logarithm of person-years as an offset variable. Incidence rates of the given disorder will be calculated for a relevant number of categories (age groups, gender, paternal and maternal age etc.). Access to the registry data has been provided and an initial study cohort has been assembled. The case–control status of the subjects will derivate for the down stream analyses according to the diagnosis under investigation (i.e. schizophrenia, autism, etc.).

### Functional studies in a case–control sample

We combined *in vivo* neuroimaging and dimensional behavioral testing to chart the biological mechanisms of 22q11 deletion. Electroencephalography (EEG) and magnetic resonance imaging (MRI) are used to measure human brain function and structure *in vivo*. Cognitive tests and psychiatric assessments are employed to provide measures of phenotypic manifestations on quantitative and qualitative scales.

The cognitive test protocol is enriched with tests, which assess neuro- and social cognitive functions that have previously been associated with 22q11 carriers [28, 29] as these impairments are also prevailing in idiopathic schizophrenia [25, 43], autism [44] or ADHD [45–47]. The specific tests are listed in Table 1. Furthermore, we use auditory mismatch negativity (MMN) paradigms in both EEG and fMRI investigations as these reveal commonly reported schizophrenia response deficiencies [48–50]. The MMN paradigm probes the brain response to a sudden sensory change, commonly tested in the auditory domain and is explained in more detail below. By applying a MMN paradigm during EEG and fMRI, we can capture specific brain responses to distinct auditory changes on a high temporal and spatial scale.

**Table 1:** The behavioral and cognitive test battery

Interview, test and questionnaires	Test outcome	Test tool	References
*Psychiatric interviews*			
MINI/MINI-KID	Psychiatric research diagnoses	Mini International Neuropsychiatric Interview Danish version 5.0.0 / Mini International Neuropsychiatric Interview for Children and Adolescents version 6.0	[53, 54, 73]
SIPS	Psychotic and prodromal symptoms	Structured Interview for Prodromal Syndromes	[55, 74]
*Cognitive abilities*			
RIST	General intelligence	The Reynolds Intellectual Screening Test	[56]
Coding test	Processing speed	Wechsler Intelligence Scale 4th ed.	[57, 58]
Letter-number sequencing test	Verbal working memory	Wechsler Intelligence Scale 4th ed.	[57, 58]
Spatial Working Memory	Spatial working memory	CANTAB^a^ (SWM)	www.cambridgecognition.com/tests/working-memory-swm
Intra-Extra Dimensional set shift	Attentional set formation, maintenance, flexibility of attention	CANTAB^a^ (IED)	www.cambridgecognition.com/tests/intra-extra-dimensional-set-shift-ied
Reaction Time	Reaction time and motor speed	CANTAB^a^ (RTI)	www.cambridgecognition.com/tests/reaction-time-rti
Emotion Recognition Task	Emotional recognition in facial expressions	CANTAB^a^ (ERT)	www.cambridgecognition.com/tests/emotion-recognition-task-ert
Rapid Visual information Processing	Sustained attention	CANTAB^a^ (RVP)	www.cambridgecognition.com/tests/rapid-visual-information-processing-rvp
Information Sampling Test	Reflection impulsivity and decision-making	CANTAB^a^ (IST)	www.cambridgecognition.com/tests/information-sampling-task-ist
Stop Signal Task	Response inhibition	CANTAB^a^ (SST)	www.cambridgecognition.com/tests/stop-signal-task-sst
TASIT, Part A2 Social Inference (minimal))	Social perception	The Awareness of Social Inference Test	[59]
B-SIT	Olfactory function	Brief Smell Identification Test	[60]
Word Selective Reminding task	Verbal memory function	Test of Memory and Learning 2nd ed.	[60]
Observations			
TOF (age 12–18 years)	Observed behavior and emotions during test situation	Test Observation Form	[63]
Questionnaires			
BRIEF (age 12–17) BRIEF-A (age 18+)	Executive functions in daily life	Behavior Rating Inventory of Executive Function	[75, 76]
ABAS-II	Adaptive functioning	The Adaptive Behavior Assessment System-Second Edition	[77]
SRS (age 12–18 years)	Social impairments, social awareness, social information processing, capacity for reciprocal social communication, social anxiety/avoidance, autistic preoccupations and traits	Social Responsiveness Scale^TM^	[78]
ADHD-RS (age 12–18 years)	Symptoms of ADHD and conduct disorders	Attention Deficit Hyperactive Disorder rating scale	[79]
SCQ (lifetime version) (W-381B)	Communication skills and social functioning	Social Communication Questionnaire lifetime form	[80, 81]
Edinburgh handedness inventory	Handedness	Edinburgh handedness inventory	[66]

^a^*CANTAB* Cambridge Neuropsychological Test Automated Battery

In addition, information from the national health registries in Denmark will be used to assess familial load of mental health issues, socio-economic and environmental risk factors and to follow the disease trajectory for each individual over time. We use an age limit of 12 years for enrollment to ensure that the children are able to comply with the entire examination.

### Recruitment

The recruitment for the functional studies has been finalized. The 22q11 deletion carriers were primarily recruited from Department of Pediatrics, Aarhus University Hospital and Department of Clinical Genetics, Copenhagen University Hospital that are the core sites in Denmark, which provide general overall supervision, care and treatment (if necessary) of 22q11 deletion carriers in Denmark. Cases were initially contacted when they had appointments at the hospital departments, or by letter and a follow-up telephone call. In addition, 22q11 deletion carriers were recruited through postings at family meetings held by the Danish National 22q11DS Association. None were recruited from psychiatric departments or any other subspecialty clinics.

Each of the recruited 22q11 deletion carriers were matched by gender and age with a non-related control subject who was randomly recruited either through *(a)* the Danish National Health Registers and contacted by letter, *(b)* public online postings at http://www.forsogsperson.dk/, or *(c)* among children of unpaid voluntary blood-donors who participate in the Danish Blood Donor Study [51]. Upon blood donation the parent (a blood donor) received a leaflet and verbal information on the project. The actual recruitment process was initiated only if the parent subsequently returned a letter of interest.

### Inclusion criteria

All subjects had to be at least 12 years old. Cases should have a clinically genetic verified 22q11 deletion based on standard clinical cytogenetic methods (Fluorescence In Situ Hybridization, Multiplex Ligation-dependent Probe Amplification, or array - Comparative Genomic Hybridization), and subsequent confirmation. Cases had a deletion within the 3-mega base region that defines the 22q11 deletion syndrome at chromosome 22q11.2. Control subjects had to be unrelated to participating case subjects and not carry the 22q11 deletion. First-degree family members in families where the 22q11 deletion is segregating (parents and full siblings) were also included if they were over 18 years of age whereas siblings, carriers of the 22q11 deletion, were included if they were over 12 years.

### Exclusion criteria

Cases should not have a deletion outside the classical 3-mega base region that defines the 22q11 deletion and should not have a diagnosis of schizophrenia or any other psychotic illnesses. Control subjects should not have a diagnosis of a) schizophrenia, schizotypal and delusional disorders (ICD10 DF20-29); b) bipolar disorder (ICD10 DF30-31); c) depression (ICD DF32-33) except for a past episode of mild or moderate depression (ICD10 DF 32.0 or 32.1); d) substance abuse; or e) have a first degree relative with a psychotic illness. Control subjects should not be family related to a case. Subjects having embodied metal fragments (i.e. acquired metal implants from surgery or had a metal brace for tooth corrections), were pregnant, or suffered from severe claustrophobia were not recruited for the MRI and fMRI sub-studies.

### Ethical considerations

The study was approved by the Regional Ethical Committee of Copenhagen, Denmark (project id: H-3-2012-136) and the Danish Data protection Agency (project id: 2007-58-0015). Access to data follows the statutory criteria given by the Danish Personal Data Protection Act governed by the Danish Data Protection Agency. Project data can be provided through personal contact to the corresponding author. All participants went through a verbal and written informed consent process. Participants under the age of 18 provided a verbal assent while their parent’s completed written consent.

### Participants

Of the 244 identified Danish 22q11 deletion carriers 136 where above the age of 12, alive, and without a schizophrenia-form disorder. Of these we were able to contact 69. In total 44 cases and 48 controls consented to participate. The characteristics of recruited subjects are shown in Table 2. The birth year distribution of the 44 22q11 deletion carriers is depicted in blue in Fig. 1. A total of five parent-offspring pairs for which the 22q11 deletion is transmitted from a parent to the child are among the recruited subjects.

**Table 2:** Demography and prevalence of previous and current mental disorder of the case–control sample recruited for the functional studies and the non-recruited 22q11 deletion carriers

	Recruited controls (*n* = 48)	Recruited 22q11 carriers (*n* = 44)	Non-recruited 22q11 carriers (*n* = 92)
Mean age (age span)	20.3 (12–48)	20.3 (12–48)	22.6(12–54)
Male:female ratio	28: 20	25:19	41:51
Caucasian ancestry ^a^	47	44	n.a.
3 megabase deletion (%)	0 (0 %)	41 (93 %)	n.a.
Segregation (%)	0 (0 %)	5 (11 %)	4 (4 %)
RIST general intelligence (S.D)^b^	107.7 (11.1)	78.9 (17.4)	n.a.
Diagnoses % (current state disorder)^c^			
Alcohol and psychoactive substance dependence	0 (0)	0(0)	1.6(n.a.)
Schizophrenia Spectrum disorders	0 (0)	0(0)	0 (n.a.)
Affective disorders	0 (0)	7 (4.5)	4.3(n.a.)
Anxiety and Fobia	(1) 9.1	11 (18.2)	8.7(n.a)
Anorexia	0(0)	0(0)	0 (n.a)
Intellectual disability	0 (n.a)	34 (n.a)	29.3(n.a)
Autism	0(n.a)	11 (n.a)	6.5(n.a)
ADHD	0 (0)	9 (11.4)	8.7(n.a)

*n.a* not assessed

^a^One control subject had Asian ancestry

^b^IQ scores are derived from the Reynolds Intellectual Screening Test (RIST) using pre-access to an updated raw- to T-score table kindly provided by the publisher (in submission)

^c^Percentages in () represents current diagnostics obtained from the M.I.N.I or M.I.N.I-kid diagnostic interviews

### Examinations

Upon inclusion all participants were evaluated using a predefined protocol that assesses psychopathology and cognitive functions in two separate sessions and include a set of self-reporting questionnaires. Each participant was invited to participate in EEG and structural and functional sub-studies. Experienced physicians and trained psychometricians conducted the psychiatric assessments and cognitive testing during home visits or at the Danish Research Center for Magnetic Resonance (DRCMR) at Hvidovre Hospital, Copenhagen, Denmark. EEG and MR examinations were conducted by trained researchers at the DRCMR. The examination of all participants has been finalized, data has been curated and data analyses are initiated.

#### Genetic assessment

All participants were screened for the presence of the 22q11 deletion using saliva samples collected after completion of the cognitive test session and on dried blood spot samples obtained from the Danish Newborn Screening Biobank [52], which contains the neonatal blood spots, routinely obtained shortly after birth of all Danes since 1982.

DNA was purified using standard protocols and the presence or absence of a deletion within the three mega base region that defines the common 22q11.2 deletion were obtained from Single-Nucleotide Polymorphism (SNP) array analyses using the HumanOmniExpressExome BeadChip (Illumina, Inc.).

#### Psychopathology and cognitive protocol

Prior to assessment each participant was carefully informed on the course and procedures for the session. The protocol included tests for the assessment of current state psychopathology, intellectual functioning, and specific social- and neurocognitive functions using; the Mini International Neuropsychiatric Interview [53] or Mini International Neuropsychiatric Interview for Children and Adolescents [54] and the Structured Interview for Prodromal Syndromes [55], the Reynolds Intellectual Screening Test [56], the Coding and Letter-Number Sequencing sub-tests from the fourth edition of the Wechsler Intelligence Scale [57, 58], the Awareness of Social Inference Test, Part A2 Social Inference (minimal) [59], the Brief Smell Identification Test [60], the Word Selective Reminding task from the second edition of Test of Memory and Learning [61], and the Tests for Spatial Working Memory, Intra-Extra Dimensional set shift, Reaction Time, Emotion Recognition Task, Rapid Visual Information Processing, Information Sampling Test and Stop Signal Task from the Cambridge Neuropsychological Test Automated Battery [62] (see Table 1). All interviews and tests were applied in the same order using a highly standardized procedure.

The consulting psychometrician immediately filled out the standardized Test Observation Form [63] for each participant age 12–21 to obtain a measure of observed behavior and emotions during the cognitive test situation. Adult participants were also asked to fill out a set of self-reported questionnaires or where appropriately assisted by their nearest caretaker. Finally, parents of participants under the age of 18 were asked to complete a set of parent-response forms for their children (See Table 1).

#### Functional brain mapping protocols

Functional brain mapping consisted of separate EEG and fMRI measurements during two types of auditory roving MMN paradigms [64] in order to study the underlying neural network of 22q11 deletion carriers compared to control subjects. In addition, the EEG protocol included an auditory steady-state gamma entrainment paradigm to study cortical integration of information. This paradigm allows to test whether 22q11 deletion carriers show a reduced entrainment of gamma band activity, similar to patients with schizophrenia [65].

The EEG and fMRI examinations were run in two separate sessions on two different days. Prior to testing each participant was interviewed about their well-being during the last 24 h (i.e. hours of sleep, intake of caffeine containing beverages and medication), tested for handedness using the Edinburgh handedness inventory [66] and subjected to two hearing tests. A standardized hearing test was used to test for the ability to detect different frequencies using an Oscilla USB-310 Tablet screening audiometer. A custom made psychophysical test assessed subjects’ subjective perception of tones between 30db and 90 db using 1–10 point likert scale (1 – cannot hear anything, 10 very loud).

### Mismatch negativity paradigm

In both EEG and fMRI, we used identical MMN paradigms, which are referred to as “roving” MMN paradigms [64]. In these roving MMN paradigms a sequence of repeated auditory standard tones was presented followed by an oddball that was randomly introduced. The oddball then became the new standard that again was followed by a new oddball stimuli [64]. We applied two different roving MMN paradigms: a “single” and a “multiplex” paradigm. In the single MMN paradigm the oddball occurred in only one auditory feature, namely frequency (pitch). Here we used either 1000Hz or 1200Hz while tones were played for 50 ms with an inter stimulus interval of 450 ms. In the multiplex MMN paradigm, an oddball occurred in frequency (Hz) and duration (ms), independently from each other. Here again, pitch oddballs occurred in either 1000Hz or 1200Hz while duration oddballs were defined as tones being played for either 50 or 100 ms with an inter stimulus interval of 450 or 400 ms respectively. Each of the MMN paradigms was applied for a total of 12 min in the fMRI setting and for 15 min in the EEG set-up.

### Gamma entrainment paradigm

In the EEG session, subjects were presented with an auditory gamma entrainment paradigm, with sequences of 40Hz click trains. Each sequence of stimulation was of 1-second duration, followed by a pause of 2 sec, and thus a stimulus onset asynchrony of 3 sec. The clicks were distributed regularly with a constant distance at a rate of 40 clicks per second each of 1 ms duration. As a control condition clicks were presented at a rate of 40Hz, but with the clicks spaced irregularly over the 1-second stimulation period, hence the control stimulus had the same physical property and only differed in how the sequence was presented, meaning that locale frequencies could deviate from 40Hz. The irregular sequence was kept constant within a session for a specific subject but differed across subjects. The two conditions were recorded during two independent blocks of 6 min each.

### Magnetic resonance imaging of the brain

Prior to scanning, subjects were carefully instructed about the experimental procedures and had to perform a test trial of the MMN paradigms to get accustomed to stimuli and task. Subjects were scanned on a Siemens Verio 3T scanner (Siemens, Erlangen, Germany) in two consecutive sessions of each 45 min with a 15 min break in between. Brain scans in the first session were acquired with a 12-channel phased-array head coil, and consisted of a 3D T2-weighted, sagittal, turbo spin echo sequence of the whole brain (TR 1 = 5000 ms; TE = 395 ms; FOV = 250 × 250; matrix = 256 × 256; GRAPPA: factor = 2; lines = 24, 176 sagittal slices with no gap, 1 mm3 voxels), followed by 2 T2*-weighted echo planar imaging fMRI scans for the two MMN paradigms (TR = 2150 ms, TE = 26 ms, flip angle = 78 °, matrix = 64 × 64; FOV = 192 × 192; 42 axial slices with 0.4 mm gap, 3 mm^3^ voxels; 345 images, 12 min). The initial four images of each fMRI scanning session were discarded to allow for equilibration of T1 signal. Additionally, we acquired two reference scans with the same sequences parameters as in the fMRI acquisition but with opposite phase encoding directions in order to correct for geometric distortions due to B0 magnetic field inhomogeneities [67].

After subjects were offered a break outside of the scanner bore, scanning was continued with a 32-channel coil. In the second session, we acquired a high-resolution three-dimensional T1-weighted, sagittal, magnetization-prepared rapid gradient echo (MPRAGE) anatomical scan of the whole brain (TR = 1900 ms, TI = 900 ms, TE = 2.32 ms, flip angle = 9°, matrix = 256 × 256, FOV = 230 × 230, 224 sagittal slices with no gap, 0.9 mm^3^ voxels), followed by whole-brain diffusion weighted imaging. Diffusion weighted imaging used a twice-refocused balanced spin echo sequence that minimized eddy current distortions [68] with ten non-diffusion-weighted images (b = 0) and 61 diffusion-weighted images (b = 1500 s/mm2) encoded along 61 independent collinear diffusion gradient orientations [68] (TR = 11,440 ms; TE = 89 ms; FOV = 220 × 220; matrix = 96 × 96; GRAPPA: factor = 2; lines = 24; 61 axial slices with no gap; 2.3 mm3 voxels). Additionally, we acquired two reference scans with the same sequences parameters as in the diffusion-weighted images acquisition but with opposite phase encoding directions in order to correct for geometric distortions due to B0 magnetic field inhomogeneities. Finally, we obtained 10 min resting-state fMRI (279 images), followed by two reference scans with opposite phase encoding directions using the same parameters as the sequences used during the MMN paradigms. During the entire time of scanning, subjects were shown a silent movie with underwater scenery free from any sudden or salient visual events in order to control for attentional load between subjects.

#### Electroencephalography

EEG data were recorded using a 128 channel ActiveTwo Biosemi System, with a sampling frequency of 4096Hz. Electrooculography and respiration pulse were recorded using external electrodes. Stimuli were delivered binaurally via insert-earphones (E-A-RTONE 3A) using the Cogent 2000 toolbox [69], running in Matlab.

Prior to the session, subjects were carefully instructed about the experimental procedures, and were presented with a couple of tones to make them comfortable with the stimuli. The EEG session was divided into five parts with a break in between each; part 1) single roving MMN, lasting 15 min, part 2) gamma entrainment paradigm lasting 6 min, part 3) irregular gamma entrainment lasting 6 min, part 4) resting state with eyes open lasting 6 min, and finally part 5) multiplex roving MMN lasting 15 min, resulting in a total time of 48 min excluding breaks. During the two roving MMN paradigms subjects were shown the same silent movie as in the scanning session. For the two gamma entrainment paradigms as well as the resting state part, subjects were asked to fixate on a fixation cross presented on a screen in front of them.

### Power considerations

Studies of genetic high-risk individuals are limited by the mutation and testing rate. However, we optimized the power for this study by investigating the most frequent mutation in humans. Although the 22q11 deletion sample size in the current study is limited we are able to utilize the Danish health registries to inform on how the recruited sample compares to the known 22q11 population as a whole. As depicted in Fig. 1 the sample that is recruited for the functional studies has a birth year distribution that resembles that of the entire Danish 22q11 population born before 2001, which reflects our random recruitment strategy. Likewise the diagnostic profile based on health registry information of the recruited 22q11 deletion carriers does not differ significantly (nominal p values >0.05) from that of the non-recruited subgroup of Danish 22q11 deletion carriers, who fulfill the same inclusion criteria as we applied to the recruited sample (12 years of age or older, alive and without schizophrenia (see Table 2, Fig. 1). Furthermore, we also take familial mental health issues into consideration. With the access to the Danish health registers we therefore have the ability to generalize findings from our sub-group of recruited individuals to the finite national case population. This is a unique and worldwide-unmatched possibility that we have in Denmark.

## Discussion

There is emerging evidence that early intervention can improve treatment outcome and reduce transition into psychosis and thus prevent some individuals from getting ill [1]. However, the challenge in psychiatry is to identify people at high risk of becoming ill and the limited treatment options.

We have established a national initiative to study the high-risk 22q11 deletion. In Denmark the majority of the 22q11 deletion carriers are known from an early age and therefore we can study them longitudinally from before a mental disorder sets in. With our initiative we are currently addressing mental health, cognitive performance and neurophysiological features among Danish 22q11 deletion carriers. This enables us to obtain more accurate information on comorbidity factors and about prodromal signs and premorbid phenotypes of psychosis and about the morbid presentation of ADHD and autism.

### Perspectives for future studies by the Danish 22q11 initiative

We trust that our initiative will grow into more extended projects where we can map molecular imprints of the 22q11 deletion and integrate this information with the data that we are currently collecting to inform on the pathogenesis of the neurodevelopmental disorders that associate with the 22q11 deletion.

Since access to relevant biological tissues is limited in humans we will turn to studies in an orthologous 22q11 deletion mouse model that has been generated within the 22q11 Initiative for translational cross species studies. It has been possible since the chromosome 22q11 region is highly conserved between man and mice [70]. Many of the traits that we have measured in the human subjects for the current study are currently being followed up in mice. Studies in mice also allow for more detailed and interventional studies than possible in humans. Therefore, mouse studies can map deficits across multiple levels from altered behavior, anatomical and physiological changes to molecular disturbances.

We will extend the molecular analyses of the human 22q11 deletion carriers that we have recruited for the current study. Our initial genetic screening for the 22q11 deletion is based on saliva samples and dried neonatal blood spots retrieved from the Danish Neonatal Screening Biobank [52]. The neonatal blood spot samples have proven well suited for extended next-generation sequencing and for metabolomic studies currently ongoing [71]. We will use these blood samples to perform extended genetic analysis to inform on additional genetic components (e.g. additional copy number variants, point mutations at the non-deleted 22q11 allele or elsewhere in the genome, imprinting etc.) that may contribute to the observed phenotype in our recruited subjects or the entire sample of known 22q11 deletion carriers in Denmark. Furthermore, the neonatal bloodspot samples are also suitable for more extended molecular profiling [72]. This opens up the possibility to provide information on the impact on mental health of early environmental exposures during prenatal development.

Early detection and enhanced treatment efficacy is required for mental health care improvements. Identification of predictive premorbid signs in 22q11 deletion carriers that can be applied by early detection teams may alter current clinical practice from treatment of manifest mental illness into targeted early interventions aimed for people at risk. These early interventions may also be applicable to symptomatic or genetic subjects at risk without known etiology. New insights into the biological disease mechanisms mandatory for novel drug developments, can improve the efficacy of the pharmacological interventions to prevent or delay the onset of mental illness not only for 22q11 deletion carriers but also for at-risk subjects without known etiology.

## Abbreviations

3D: Three dimensional
3T: Three tesla
ABAS-II: The Adaptive Behavior Assessment System-Second Edition
ADHD: Attention deficit hyperactivity disorder
ADHD-RS: Attention Deficit Hyperactive Disorder rating scale
B0: The main magnetic field
BRIEF: Behavior Rating Inventory of Executive Function
B-SIT: Brief Smell Identification Test
CANTAB: Cambridge Neuropsychological Test Automated Battery
EEG: Electroencephalography
fMRI: Functional magnetic resonance imaging
FOV: Field of view
GRAPPA: Generalized Autocalibrating Partially Parallel Acquisition
Hz: Hertz
ICD: International Classification of Diseases system
IQ: Intelligence quotient
MINI: Mini-International Neuropsychiatric Interview
MINI-KID: Mini International Neuropsychiatric Interview for Children and Adolescents
MMN: Mismatch negativity
MRI: Magnetic resonance imaging
ms: Milliseconds
RIST: The Reynolds Intellectual Screening Test
SCQ: Social Communication Questionnaire lifetime form
SIPS: Structured Interview for Prodromal Syndromes
SRS: Social Responsiveness Scale
T1: The longitudinal relaxation of the net magnetization vector
T2: The transverse relaxation of the net magnetization vector
T2*: Decay of transverse magnetization caused by a combination of spin-spin relaxation and magnetic field inhomogeneity
TASIT: The Awareness of Social Inference Test
TE: Echo time
TOF: Test Observation Form
TR: Repetition time
